# Glances at the Hospitals

**Published:** 1903-08-29

**Authors:** 


### GLANCES AT THE HOSPITALS.

### ROYAL HOSPITAL FOR SICK CHILDREN, GLASGOW.

The ordinary income for the year was £5,563, and the ordinary expenditure £5,942, there being a deficiency on the year's working of about £379. The extraordinary income amounted to £6,394. Of this sum upwards of £3,000 was transferred to capital account, and £379 was applied to wipe off the adverse balance on the maintenance account. On January 1st the hospital contained 63 children; 854 were admitted during the year, making a total of 917 under treatment. Of these 336 were cured, 234 improved, 152 not improved, 122 died, and 73 remained in the hospital. The highest number resident was 79; the lowest was 27; the average daily number resident was 67; the average duration in hospital was 27 days. The percentage of deaths was 14·45 of the total number discharged; but 33 died within 48 hours of admission. The daily cost of each patient was a fraction under 3s. 9d.; the total cost of each patient was £5 3s. 9d.; and the cost of each bed occupied was £68 6s. The dispensary cases amounted to 9,109, and involved 32,246 attendances. These figures show that a large amount of useful work is being done by the hospital, and the Governors point out that it is the only one of its kind within reach of two millions of inhabitants. We notice that 612 of the patients were sent from places within the municipal boundaries, 97 from the suburbs, and the rest from outlying districts. When it is remembered that Edinburgh has a Sick Children's Hospital with 120 beds, and Aberdeen one with 75, it is not without cause that the Governors state that what is wanted in Glasgow is a new hospital built on a healthy site, with all modern improvements, and capable of accommodating 200 children. Glasgow has a population of more than half a million; but this should be a West of Scotland undertaking, and a very small contribution per head would raise the sum required, say, £80,000 or £100,000. The medical and surgical tables are very well arranged, and give every essential particular. There is also a table showing the causes of death in 77 cases, and an operation table having columns for the numbers, the cured, the relieved, and the deaths. The total number of cases operated on was 314, and of these 158 were cured, 121 relieved, and 35 died.

### DERBYSHIRE ROYAL INFIRMARY.

The income amounted to £9,273, and the expenditure to £9,753, showing a deficiency of £480. At the beginning of the year there were 158 patients in residence and 2,090 were admitted since, making a total of 2,248. Of these 1,411 were discharged recovered, 424 were discharged relieved, 84 were discharged for various reasons, 127 died, and 161 remained under treatment. The cost of each bed occupied was £53 15s. 6d., the average cost of each in-patient was £3 15s. 7d., the average cost of each in-patient per week was £1 0s. 8d., and the out-patients cost 2s. 6d. each. The average stay in the hospital was 25 days. The medical and surgical tables are carefully drawn out, and show columns for the numbers cured, relieved, unrelieved, and died; and there is another column for remarks. We find that of 47 cases of enteric fever 38 were cured and 9 died. In 5 of these toxæmia was the cause of death, in 2 perforation, and in 2 pneumonia. There were 26 cases of acute rheumatism and all recovered, although many were complicated. Ovariotomy was performed 17 times, recovery following in 16 and death in 1. Appendectomy was performed 15 times, all being successful. In 67 cases of herniotomy for strangulated hernia there were 62 recoveries and 5 deaths. The anæsthetic table shows that ether was given 637 times, chloroform 474 times, chloroform and ether 55 times, nitrous oxide 76 times alone, with ether 57, cocaine locally in 165 cases, and anæstile 20. From the above "Glance" it will be seen that the report of this infirmary is unusually well arranged and instructive.

### NORTH HERTS AND SOUTH BEDS HOSPITAL.

The income amounted to £1,322, and the expenditure to £1,367, the latter exceeding the former by £45. There were 14 patients in residence at the commencement of the year, and 230 were admitted during it, making a total of 230. The average stay in the hospital was 25 days. The medical and surgical tables show the total numbers under treatment for the various diseases; the numbers cured, relieved, incurable, died, and the number remaining in the wards. A table of the operations is printed, but here results are not shown, and nothing is said as to the nature of the anæsthetics used.

